# Chitosan Nanoparticles Loaded Poloxamer 407 Gel for Transungual Delivery of Terbinafine HCl

**DOI:** 10.3390/pharmaceutics14112353

**Published:** 2022-10-31

**Authors:** Kamran Hidayat Ullah, Faisal Rasheed, Iffat Naz, Naveed Ul Haq, Humaira Fatima, Nosheen Kanwal, Tofeeq Ur-Rehman

**Affiliations:** 1 Department of Pharmacy, Quaid-i-Azam University, Islamabad 45320, Pakistan; 2 Patient Diagnostic Lab, Isotope Application Division (IAD), Pakistan Institute of Nuclear Science and Technology (PINSTECH), Nilore, Islamabad 45650, Pakistan; 3 Department of Biology, Science Unit, Deanship of Educational Services, Qassim University, Buraidah 51452, Saudi Arabia; 4 Department of Biochemistry, Science Unit, Deanship of Educational Services, Qassim University, Buraidah 51452, Saudi Arabia

**Keywords:** chitosan nanoparticles, poloxamer 407 gel, transungual drug delivery, onychomycosis, face-centered central composite design

## Abstract

The current study aimed to develop chitosan nanoparticles (CSNP) loaded poloxamer 407 (P407) gel formulation for transungual delivery of terbinafine HCl (TBN). TBN-CSNP were prepared by nanoprecipitation method and optimized by face-centered central composite design (FCCCD). Optimized TBN-CSNP formulation exhibited a spherical shape with hydrodynamic diameter; zeta potential and entrapment efficiency (EE) of 229 ± 5 nm; 37 ± 1.5 mV; and 75 ± 2% respectively. The solid state of TBN and its compatibility with formulation ingredients were confirmed through XRD and FTIR analysis respectively. TBN-CSNP loaded P407 gel exhibited pseudoplastic rheological behavior having a spreadability of 11 ± 2 g·cm/s. The washability study showed that 40 ± 2% of the gel was eroded after washing 12 times. Drug release from TBN-CSNP- and TBN-CSNP-loaded gel was 84 ± 5% and 57 ± 3%, respectively. The cumulative quantity of TBN permeated from TBN-CSNP-loaded P407 gel and TBN-loaded P407 gel was 25 ± 8 and 27 ± 4 µg/cm^2^, respectively. The nail uptake study showed that 3.6 ± 0.7 and 2.1 ± 0.3 µg of rhodamine was uptaken by the nail following 2 h topical application of TBN-CSNP loaded P407 gel and TBN loaded P407 gel, respectively. Hence, the developed CSNP-based P407 gel formulation can be a potential carrier for transungual delivery of TBN to topically treat onychomycosis.

## 1. Introduction

Onychomycosis is one of the highly prevalent nail disorders and accounts for 50% of all nail diseases. It affects 10% of the general population and mostly affects people of age over 60 years, diabetic patients, and human immunodeficiency virus disease [1,2]. It is commonly caused by *Trichophyton rubrum*, *Trichophyton mentagrophyte*, and *Candida albicans* [3]. Characteristics of onychomycosis include discoloration of the nail, increased thickness of the nail plate, onycholysis, and pain associated with infection [4,5]. The current treatment of onychomycosis involves the use of antifungal drugs systemically or topically. However, the use of oral therapy is limited due to liver toxicity, prolonged time required for treatment, drug interactions, expensive medication, increased chances of fungal resistance, and recurrence of fungal infection [6]. Topical drug delivery is preferred because of its better safety profile, direct application to the infection site, and relatively lower cost of therapy which lead to better patient compliance [7]. Unfortunately, the success rate of topical therapies is limited due to poor penetration of antifungal agents into the nail plate. In addition, easy removal or washing of conventional topical formulations while doing daily routine activities necessitates their frequent application, which leads to poor patient compliance. This creates a need to develop novel formulations that provide efficient transungual delivery and are capable of sustainably eradicating the fungus in the nail.

The prominent strategies used for transungual delivery of terbinafine (TBN) to topically treat onychomycosis include the utilization of iontophoresis [8], a nano-vesicular system [9], and an in-situ film-forming system over the nail plate [10]. However, iontophoresis is invasive and requires high voltage for the delivery of the drug [11]. Similarly, film-forming agents need organic solvents for their removal from the nail surface after use [12].

Polymer-based platforms have gained much attention and have been utilized in different biomedical applications [13]. A previous study demonstrated the potential of polymeric nanoparticles to create a drug reservoir in the nail plate for its transungual delivery [14]. Transungual delivery utilizing nanoparticles promotes deeper penetration of the drug into the nail plate, enhances retention in the nail plate, and controls the release of the drug, which ensures drug release at the infection site for an extended duration to maintain the desired therapeutic drug concentration [15,16,17]. Also, nanoparticles can find their way through periungual skin to release the active substance, which will spread laterally to the nail bed [18].

Different natural and synthetic polymers are used in the preparation of nanoparticles among which chitosan, is of special importance. Chitosan is a widely used polymer in biomedical applications due to its biocompatibility, biodegradability, mucoadhesion, and antimicrobial characteristics [19,20]. Its capability to alter keratin structure [21], greater binding ability to negatively charged nail keratin because of its positive charge, and its humectant property causing enhanced hydration of the nail plate [22] make it a suitable candidate for transungual delivery of drugs. In addition, chitosan possesses intrinsic antifungal activity, which is said to be further enhanced when formulated into nanoparticles [23]. We hypothesize that the application of TBN-loaded chitosan nanoparticles (TBN-CSNP) will create an antifungal reservoir in the upper dorsal layers of the nail plate for extended release and better penetration of TBN into lower layers of the nail plate and nail bed. However, to penetrate the nail plate, the nanoparticles should be available for sufficient time at the nail surface. Aqueous suspension of nanoparticles has poor retention at the nail surface [24], which can be improved by incorporating it into a polymer-based gel formulation. Poloxamers 407 (P407) belongs to a group of poly (ethylene oxide)–poly (propylene oxide)–poly (ethylene oxide) (PEO–PPO–PEO) amphiphilic triblock copolymers and the high water content of its gel may hydrate the nail plate, which will ultimately facilitate the permeation of drugs across the nail plate. P407 gel can also prolong the retention time of the nanoparticles at the nail surface [13]. Therefore, the current study aimed to develop TBN-CSNP and incorporate it into a P407-based gel to enhance the transungual delivery of TBN.

## 2. Materials and Methods

### 2.1. Materials

Terbinafine HCl was gifted by Max pharma, Islamabad. Chitosan (molecular weight 50–190 kDa, degree of deacetylation >76%, viscosity 122 cP), poloxamer P407, and thioglycolic acid were purchased from Sigma-Aldrich, Dorset, UK through a local supplier (United Traders, Islamabad, Pakistan). Lecithin and Tween 80 was purchased from Merck, Darmstadt, Germany through United Traders, Islamabad, Pakistan. Glacial acetic acid was supplied by Labscan limited, Bangkok, Thailand through United Traders, Islamabad, Pakistan. Ethanol was purchased from BDH Laboratory supplies, Dorset, UK through United Traders, Islamabad, Pakistan. Cellulose membrane was purchased from Merck, Germany through United Traders, Islamabad, Pakistan.

### 2.2. Preparation of Terbinafine HCl Loaded Chitosan Nanoparticles

The nanoprecipitation method was used to prepare TBN-CSNP [25]. Briefly, accurate amounts of TBN (20 mg) and lecithin (10 mg) were dissolved in ethanol and injected dropwise into the aqueous acetic acid solution (1% *v*/*v*, 10 mL), containing tween 80 and chitosan under stirring. The resulting dispersion was kept on stirring (800 RPM) overnight at room temperature to evaporate ethanol. The transparent pellet of nanoparticles along the walls of the tube was collected after centrifugation at 13,500 rpm at 4 °C for 1 h washed with distilled water three times, and lyophilized to obtain a dry powder of nanoparticles.

### 2.3. Optimization of Nanoparticles by Design Expert

Response surface methodology (RSM) based on face-centered central composite design (FCCCD) was applied to examine the relationship of independent factors and response variables which involved full factorial with five replicates in the center. RSM is used to identify significant variables and optimum process parameters and investigate the interaction between independent variables and response variables with a smaller number of experiments. A two-factor three-level central composite design was used which consist of 13 different runs of experiments with four replicates in the center generated by Design-Expert^®^ 11.1.2 software (State Ease Inc., Minneapolis, MIN, USA). The concentration of chitosan (X1) and tween 80 (X2) were the independent variables while the responses studied were particle size (Y1) and EE (Y2). The goal of optimization was to keep particle size minimum and EE maximum.

### 2.4. Characterization of Chitosan Nanoparticles

#### 2.4.1. Percent Yield, EE (%), and Drug Loading (%)

The percent yield of optimized nanoparticle formulation was determined on the basis of the total amount of TBN, chitosan, and excipient used.

The percent production yield was calculated using the following equation

$$\mathrm{Yield}\;\left(\%\right)=\frac{\mathrm{Weight}\;\mathrm{of}\;\mathrm{CSNP}}{\mathrm{Total}\;\mathrm{weight}\;\mathrm{of}\;\mathrm{TBN}+\mathrm{Chitosan}}\times 100$$

EE of optimized formulation was determined by analyzing TBN in the external aqueous phase during the preparation of nanoparticles as previously reported [25]. Briefly, the prepared TBN-CSNP were centrifuged at 13,500 rpm for 1 h at 4 °C, and then, the supernatant was analyzed for quantity of TBN using HPLC. The entrapped drug in the TBN-CSNP was obtained by subtracting the remaining amount of TBN in the supernatant from the total quantity of the TBN used to prepare the TBN-CSNP using the following equation [26].

$$\mathrm{Entrapment}\;\mathrm{efficiency}\;(\%)=\frac{\mathrm{Total}\;\mathrm{Drug}\;\mathrm{used}\;-\;\mathrm{Free}\;\mathrm{drug}}{\mathrm{Total}\;\mathrm{drug}\;\mathrm{used}}\times 100$$

The formula for calculating drug loading is as follows [26]:

$$\mathrm{Drug}\;\mathrm{loading}\;\left(\%\right)=\frac{\mathrm{Amount}\;\mathrm{of}\;\mathrm{drug}\;\mathrm{in}\;\mathrm{CSNP}}{\mathrm{Total}\;\mathrm{weight}\;\mathrm{of}\;\mathrm{CSNP}}\times 100$$

#### 2.4.2. Particle Size and Zeta Potential Analysis

Particle size and zeta potential of prepared TBN-CSNP were determined using a zeta-sizer (Malvern, Nano ZS-90, Worcestershire, UK) at 25 ± 2 °C. Freshly prepared TBN-CSNP suspension was 100 times diluted with distilled water and analyzed.

#### 2.4.3. Morphology of Chitosan Nanoparticles

The external morphology of nanoparticles was examined by utilizing SEM. Lyophilized powder of optimized TBN-CSNP was gold sputtered in a sputter coater (SPI-MODULE, SPI Supplies, West Chester, PA, USA) for 90 s at 30 mA and observed at various magnifications. A scanning electron microscope (Joel JSM-5910, Joel Ltd., Tokyo, Japan) was used at the voltage of 15 keV to take images at different resolutions.

#### 2.4.4. Drug Polymer Interaction

Drug-polymer compatibility was confirmed by Fourier transform-infrared (FTIR) spectroscopy. The FTIR spectra of TBN, chitosan, physical mixture, and TBN-CSNP were obtained using FTIR-spectrophotometer (Spectrum 65, Perkin Elmer, MA, USA). Pellets were prepared with KBr discs in a ratio of 1:100 and force was applied for several minutes to obtain the uniform pellets. These pellets were placed in between two plates in a sample holder and scanned at wavelengths ranging from 4000 to 515 cm^−1^. The absorbance was plotted against their corresponding wavenumber.

#### 2.4.5. Solid State Characterization

X-ray diffractometry was performed to identify the crystalline or amorphous nature of the formulation ingredients. Drugs, polymers, and the final formulations were scanned using an X-ray diffractometer (Philips X’ Pert PRO 3040/60) operating at an angle of 2θ in the range of 20° to 80°.

#### 2.4.6. Stability Study

Stability studies of the TBN-CSNP were carried out according to ICH guidelines at 4 °C, 25 °C, and 40 ± 2 °C ± 5% for a time period of 6 months. TBN-CSNP were checked for their particle size and zeta potential after 0, 1, 3, and 6 months while the amount of loaded drug was determined after 0, and 6 months to check the degradation of the TBN in TBN-CSNP [27].

### 2.5. Preparation of Poloxamer 407 Gel

The P407 gel was prepared by adding P407 (27.32% *w*/*w*) and TGA (4.72% *w*/*w*) to water and then kept at 4–5 °C until a homogenous solution was obtained. TBN-CSNP loaded P407 gel formulation was prepared by mixing CSNP (equivalent to 10 mg TBN) with a sufficient quantity of P407 gel solution to produce 1 g of the final gel formulation at 4 °C. The solution was warmed until a smooth gel was produced [28,29]. TBN-loaded gel and TBN-loaded P407 gel (without TGA) were also prepared [13].

### 2.6. Characterization of Chitosan Nanoparticles Loaded Poloxamer 407 Gel

#### 2.6.1. Organoleptic Evaluation and pH

TBN-CSNP-loaded P407 gel was evaluated for organoleptic properties. The organoleptic evaluation was conducted by observing the color, clarity, phase separation, and formation of precipitates visually.

The pH was determined by dissolving 1 g of gel in 100 mL of distilled water and evaluated for pH using a digital pH meter (Eutech pH 700 Eutech Instruments, Singapore) [30].

#### 2.6.2. Rheology

The rheology of the TBN-CSNP-loaded P407 gel formulations was determined using a Brookfield rheometer (DV3T, Middleboro, MA, USA). Viscosity was determined at 32 ± 2 °C using spindle CPA-52Z (Model DV3T, Brookfield Engineering Laboratories, Middleboro, MA, USA). The graph of apparent viscosity (η) to the function of shear rate (s-1) was plotted to observe the flow behavior of the prepared gel.

#### 2.6.3. Spreadability

For the determination of spreadability, 1 g of the gel was placed on a clean glass slide. Another glass slide was placed over this slide followed by the placement of a weight equivalent to 500 g on the upper glass. After 1 min, the weight was removed, and the spreading diameter was recorded [31].

The following equation was used to calculate the spreadability [32]:

S = (m × l)/t

where S is the spreadability of the gel (g·cm/s), m is the weight applied (g), l is the diameter of the gel (cm), and t is the time for which the weight is applied (s).

#### 2.6.4. Washability/Erosion Profile

The erosion profiles were determined by the gravimetric method [33] with slight modification. Briefly, 1 g of TBN-CSNP loaded P407 gel formulation (equivalent to 10 mg of TBN) was added into a small glass tube, which was kept in a beaker containing water maintained at 32 °C. Phosphate buffer pH 5.5 (3 mL) equilibrated at 32 °C was added into the glass tube. At regular intervals, the phosphate buffer was removed, and a reduction in the weight of the P407 gel was noted to determine the amount of eroded gel. One cycle of addition of solvent and removal was considered as one washing and the number of washings required to remove the percentage of P407 gel from the glass tube was noted.

#### 2.6.5. In Vitro Drug Release from Chitosan Nanoparticles and Nanoparticles Loaded Poloxamer 407 Gel

In vitro release profile of TBN-CSNP formulation and TBN-CSNP loaded P407 gel formulation was studied by dialysis membrane diffusion model [34]. Briefly, TBN-CSNP formulation and TBN-CSNP loaded P407 gel formulation (equivalent to 5 mg) were added separately into cellulose membrane (12–14 kDa, Merck, Darmstadt, Germany) and 3 mL of release medium was added to it. Formulation-containing membranes were then placed in separate 50 mL of release medium PB (pH 5.5):ethanol (9:1 ratio) keeping the temperature at 32 °C and stirred using a magnetic stirrer. At predetermined intervals, the release medium from the beaker was withdrawn and replaced with the fresh release medium to maintain the sink condition. The samples were analyzed for the quantity of TBN at 283 nm and released using a UV spectrophotometer (Halo DB 20, Dynamica, Victoria, Australia) [35]. Similarly, in vitro release from TBN suspension was also performed.

#### 2.6.6. Kinetic Modelling of In Vitro Drug Release

Kinetic models were applied to predict the release kinetics and mechanism of drug release by applying mathematical models, that is, Zero order, First order, Higuchi, and Korsmeyer–Peppas models using the DD solver Microsoft Excel add-in program. The most suitable model was selected based on the goodness of fit test (calculation of R^2^). The *n* value obtained from Korsmeyer–Peppas model was used to find the mechanism of drug release.

#### 2.6.7. Ex Vivo Permeation

An ex vivo permeation study across human nails was conducted using a Franz diffusion cell (Permegear, Pennsylvania, USA). Briefly, nail clippings obtained from healthy female volunteers of age between 20–30 years were washed with distilled water and allowed to air dry. The nail clippings were then trimmed into a dimension of 3 × 3 mm and placed between the donor and receiver compartment and their interface was occluded with parafilm. Phosphate buffer (pH 5.5):ethanol (9:1) was added to the receiver compartment and maintained at 32 °C. The TBN-CSNP-loaded P407 gel formulation (equivalent to 1 mg of TBN) was put on the nail surface. Samples were taken at regular intervals and quantified at 283 nm using a UV spectrophotometer. The cumulative quantity of the TBN (µg) permeated per unit area (cm^2^) of the nail was plotted versus time (h). The same experiment was also performed for the TBN-loaded P407 gel without TGA.

#### 2.6.8. Nail Uptake Efficiency

To determine the uptake efficiency of formulations and creation of drug reservoir in the nail plate, rhodamine-B-loaded CSNP were prepared according to the method mentioned in Section 2.2, While CSNP-loaded P407 gel, rhodamine-B-loaded P407 gel and rhodamine-B-loaded P407 gel (without TGA) were prepared using the method mentioned in Section 2.5. Prepared formulations were applied on separate nail clippings maintained at 32 °C. After 2 h, formulations from the nail surface were removed, rinsed briefly with ethanol, and dried with a clean tissue. The nail samples were incubated with a solution mixture of isopropanol: 0.1 M sulfuric acid (4:1) at 60 °C for 1 h to extract the rhodamine B taken up by the nail tissue. The extracted solutions were analyzed for rhodamine B content at 556 nm using a UV-visible spectrophotometer [36].

#### 2.6.9. Statistical Analysis

All experiment results are expressed as mean ± standard deviation (S.D). The number of experiment replicates is shown in the captions of the figures. Statistical significance was determined using the Student’s t-test and analysis of variance (ANOVA). p value of less than 0.05 was considered statistically significant.

## 3. Results

### 3.1. Effect of Independent Variables on Particle Size of TBN Loaded Chitosan Nanoparticles

Different formulations of TBN-CSNP (13 runs given by the FCCCD using Design Expert^®^ 11.1.2 software) depicted varied hydrodynamic diameters; in the range of 139 ± 2 to 347 ± 16 nm (Table 1). Analysis of variance (ANOVA) was applied to develop the polynomial equation of the response variables. F value of 19.6 and p value of less than 0.05 indicated that the quadratic model can be applied to evaluate the impact of independent variables on the size of TBN-CSNP. The “Predicted R^2^” of 0.72 was close to the “Adj R^2^” of 0.89. The coefficient of variation (CV) for the quadratic model was 9.3%. The “Adequate Precision” value of 14.74 indicated adequate signal. The F value for Lack of Fit was 0.63, which showed an insignificant lack of fit.

Figure S1 (Supplementary Materials) depicts that predicted and experimental values were close to each other indicating smaller residuals and a lack of significant error. Figure S2 (Supplementary Materials) shows the closeness of residuals to a straight line, which confirmed that residuals were normally distributed.

**Figure 1:**
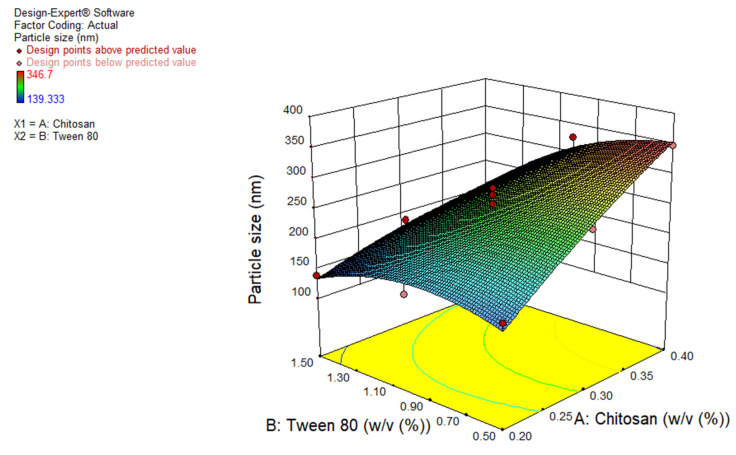
3D surface plots showing effect of concentration of chitosan and tween 80 on size of TBN-CSNP.

A *p* value of less than 0.05 indicates that A, B, AB, and B^2^ were significant model terms. The polynomial equation was:

Y_1_ = +253.3 + 67.58A − 41.4 B − 32.3AB − 10.60A^2^ − 32.2B^2^

where A and B are the concentrations (% *w*/*v*) of Chitosan and tween 80, respectively. The positive sign shows direct and the negative sign shows an inverse relationship between the independent variable and response. The polynomial equation generated for particle size (Y1) demonstrated that both chitosan concentration (regression coefficient = 67.6, F value = 57.9) and tween 80 (regression coefficient = −41.4, F value = 21.7) have a significant effect on particle size. Increasing the concentration of chitosan significantly increased (*p* < 0.05) the particle size (Table 1 and Figure 1). However, a significant reduction in particle size (*p* < 0.05) occurred when the concentration of tween 80 was increased (Table 1 and Figure 1).

### 3.2. Effect of Independent Variables on Entrapment Efficiency of TBN Loaded Chitosan Nanoparticles

The EE of 13 different runs of TBN-CSNP were found in the range of 49 ± 3 to 81 ± 0.3%. F value of 24.9 and *p* value of less than 0.05 depicted that the quadratic model can be applied to evaluate the impact of independent variables on EE (Table 1). The “Predicted R^2^” of 0.85 was close to the “Adjusted R^2^” of 0.91. CV for the quadratic model was 4.6%.

“Adequate Precision” of 13.2 indicated adequate signal. The F value of 0.25 for Lack of Fit depicted insignificant Lack of Fit. As shown in Figure S3 (Supplementary Materials), predicted and experimental values were close to each other indicating smaller residuals and a lack of significant error. Figure S4 (Supplementary Materials) shows the closeness of residuals to a straight line, which confirmed that residuals were normally distributed.

**Figure 2:**
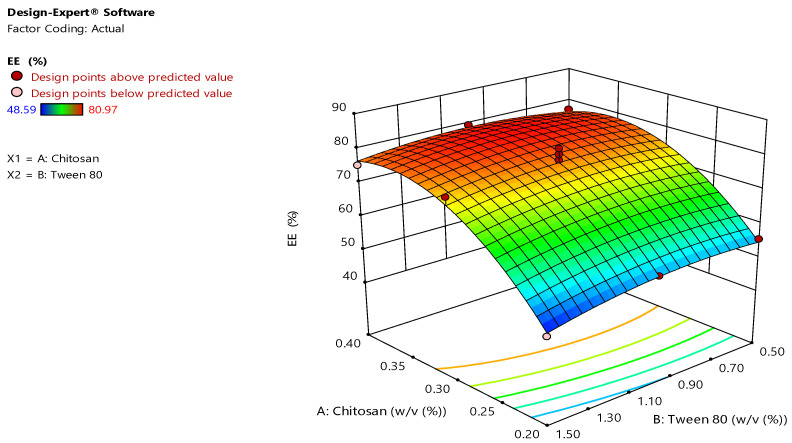
3D surface plots showing effect of chitosan and tween 80 concentration on the EE of TBN-CSNP.

A, B, AB, and B^2^, A, A^2^ were significant model terms (*p* < 0.05). The equation fitted to the data was:

Y_2_ = +77.00 + 12.18A − 1.26B + 1.26AB − 10.30A^2^ − 2.40B^2^

where A and B show the concentrations (% *w*/*v*) of chitosan and tween 80, respectively. Polynomial equation for Y2 showed that effect of chitosan concentration was significant (regression coefficient = 12.2, F value = 83.5) on EE while effect of tween 80 was insignificant (regression coefficient = −1.26, F value = 0.89). Increasing concentration of chitosan significantly increased (*p* < 0.05) the EE (%) (Table 1 and Figure 2). However, an insignificant reduction (*p* > 0.05) in EE was observed when the concentration of tween 80 was increased (Table 1 and Figure 2).

### 3.3. Response Optimization of Chitosan Nanoparticles

Optimized TBN-CSNP formulation with minimum size and maximum EE was selected after numerical optimization. The optimized formulation contains 0.32% chitosan, and 1.44% tween 80. Values of hydrodynamic diameter and EE predicted by the quadratic model were 197 nm and 75.8%, respectively. An optimum formulation was prepared, and its responses were observed to validate the model. The experimental value of hydrodynamic diameter and EE of the optimized TBN-CSNP formulation was 229 ± 5 nm and 75 ± 2%, respectively, which correlated well with the values predicted by the quadratic model.

### 3.4. Characterization of Chitosan Nanoparticles

#### 3.4.1. Percent Yield, EE, and Drug Loading

Percent yield, EE, and drug loading of the optimized formulations were 64 ± 5%, 75 ± 2%, and 18 ± 1% respectively.

#### 3.4.2. Particle Size and Zeta Potential

The average hydrodynamic diameter of the TBN-CSNP was 229 ± 5 nm when the optimized quantities of chitosan and tween 80 were used. The Zeta potential observed for TBN-CSNP was 37 ± 1.5 mV.

#### 3.4.3. Surface Morphology by SEM

**Figure 3:**
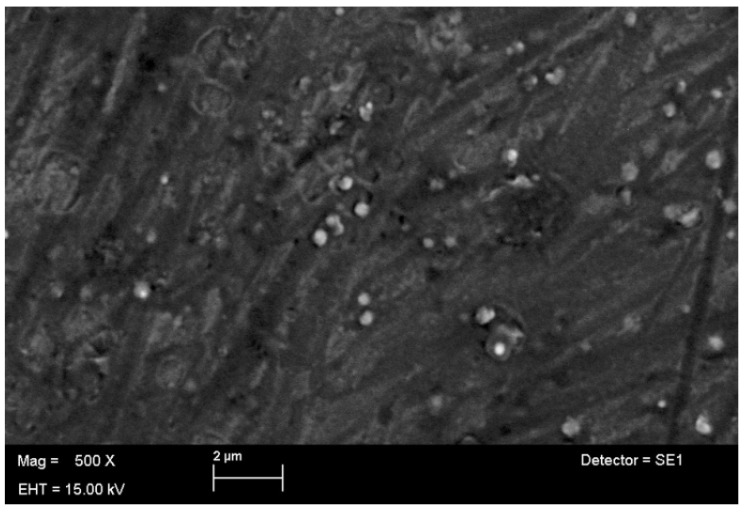
SEM images of optimized TBN-CSNP.

SEM images of the TBN-CSNP at different magnifications are shown in Figure 3. The shape of the TBN-CSNP was spherical, with smaller particle size, smooth surface, and uniform distribution.

#### 3.4.4. Drug Polymer Interaction

**Figure 4:**
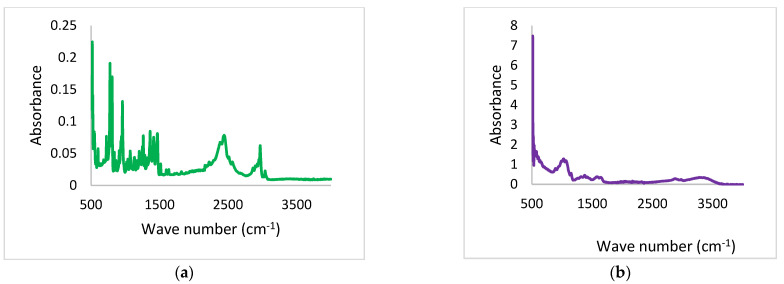

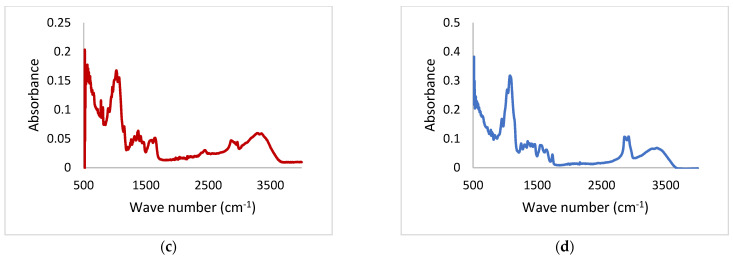
FTIR spectra of (**a**) pure drug, (**b**) polymer (**c**) Physical mixture, and (**d**) TBN-CSNP.

The FTIR analysis of TBN, Chitosan, physical mixture, and TBN-CSNP is displayed in Figure 4. TBN showed a characteristic functional group peak at the wavenumber of 3012 cm^−1^ (alkenyl C-H stretch), 2921 cm^−1^ (alkyl C–H stretch), and 2223 cm^−1^ (alkynyl C≡C stretch). Chitosan showed a peak at 3348 cm^−1^ for both O-H and N-H stretch, 2867 for C-H stretching vibration, 1643 for amide (CONH_2_), and 1072 for stretching vibration of C-O.

FTIR spectra of the physical mixture showed characteristic absorption bands of TBN at 3012, 2923, and 2221 cm^−1^ while the absorption band of chitosan was observed at 3348, 2869 1640, and 1070 cm^−1^. FTIR spectra of TBN-CSNP formulation showed the absorption bands of TBN at 3010 cm^−1^, 2220 cm^−1^, and 2921 cm^−1^. Characteristic peaks of chitosan were observed at 3361 cm^−1^, 2860, 1640, and 1072 cm^−1^. This confirmed that no chemical interaction occurred between TBN and polymer.

#### 3.4.5. Solid State Characterization

**Figure 5:**
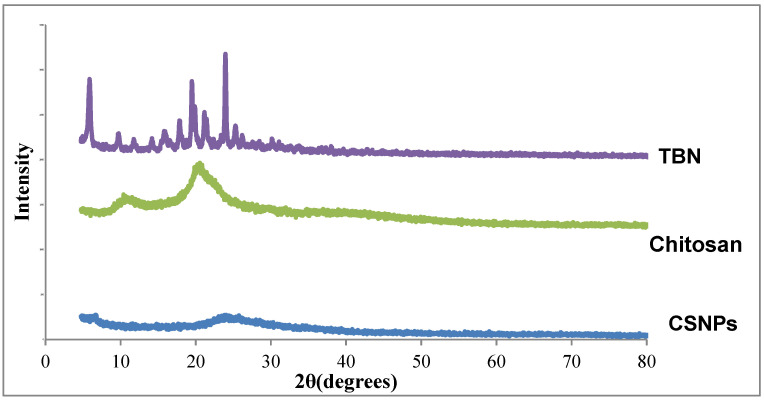
XRD Diffractrogram of TBN, Chitosan, and TBN-CSNP.

As TBN is crystalline in nature so the diffraction spectrum depicted sharp and intense peaks for TBN whereas, chitosan showed a peak at 10.38°, which corresponds to the hydrated crystalline form. The peak at 20.83° corresponds to the regular crystal lattice of the chitosan. These peaks confirmed the hydration and semicrystalline nature of chitosan (Figure 5). The spectrum of CSNP showed the disappearance of the hydrated crystalline peak (10.38°) and a reduction in the intensity of the peak at 20.83°, which confirmed the reduction in the crystallinity and amorphous nature of prepared TBN-CSNP.

#### 3.4.6. Stability of Chitosan Nanoparticles

As shown in Table 2, the stability of the formulated nanoparticles was maintained well at 5 ± 3 °C without any change in the hydrodynamic diameter, zeta potential, and drug content. A minor increase in the particle size occurred at 25 ± 2 °C, which further increased at 40 ± 2 °C. The zeta potential did not show significant changes and nanoparticles persist in the charge to prevent their aggregation. A minor decrease in the loaded quantity of TBN was observed after 6 months.

### 3.5. Characterization of Chitosan Nanoparticles Loaded Poloxamer 407 Gel

#### 3.5.1. Organoleptic Evaluation and pH

The prepared gel was colorless, translucent, and smooth to touch having no grittiness. The pH of TBN-CSNP-loaded P407 gel formulation was found to be 5 ± 0.2.

#### 3.5.2. Rheological Behavior and Spreadability

**Figure 6:**
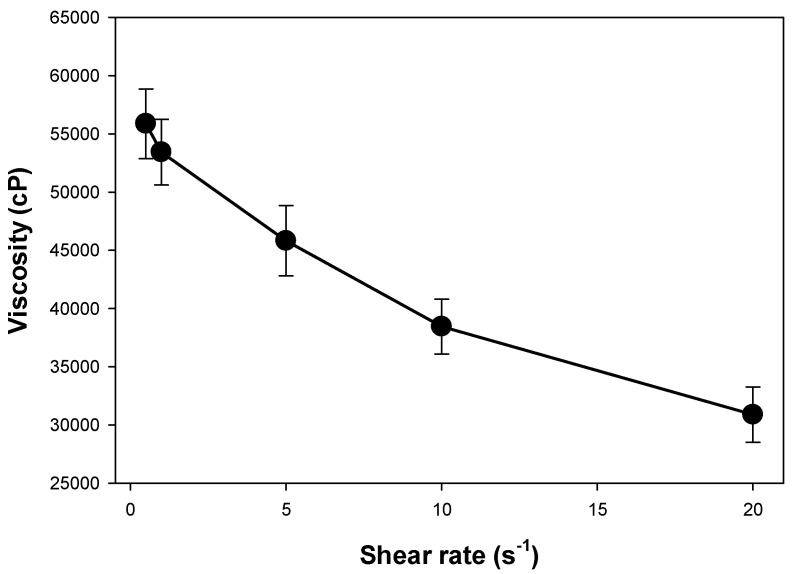
Apparent viscosity (η) vs shear rate of TBN-CSNP loaded P407 gel. Each value represents the mean ± S.D (*n* = 3).

The graph presented in Figure 6 showed that an increase in shear rate causes a decrease in the apparent viscosity of the gel. This demonstrated the non-Newtonian (pseudoplastic) behavior of the formulated gel. Spreadability values for the TBN-CSNP loaded P407 gel was found to be 11 ± 2 g·cm/s.

#### 3.5.3. Washability/Erosion Profile

**Figure 7:**
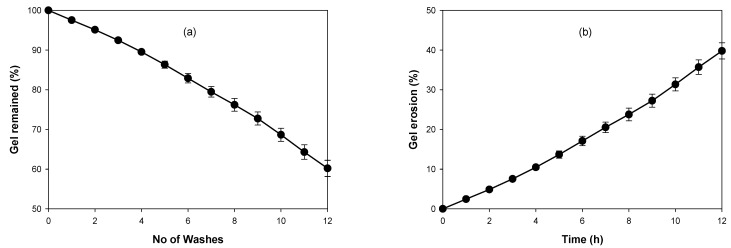
(**a**) Washability and (**b**) gel erosion profile of TBN-CSNP loaded P407 gel in phosphate buffer pH 5.5. (Each value represent mean ± S.D (*n* = 3).

The washability study showed that 83 ± 1% of the TBN-CSNP-loaded P407 gel was retained after six washings while 17 ± 1% of the gel was eroded (Figure 7). The remaining amount of the gel after 12 washings was 60 ± 2% while the amount of eroded gel was 40 ± 2%.

#### 3.5.4. In Vitro Drug Release from Chitosan Nanoparticles and Nanoparticles Loaded Poloxamer 407 Gel

**Figure 8:**
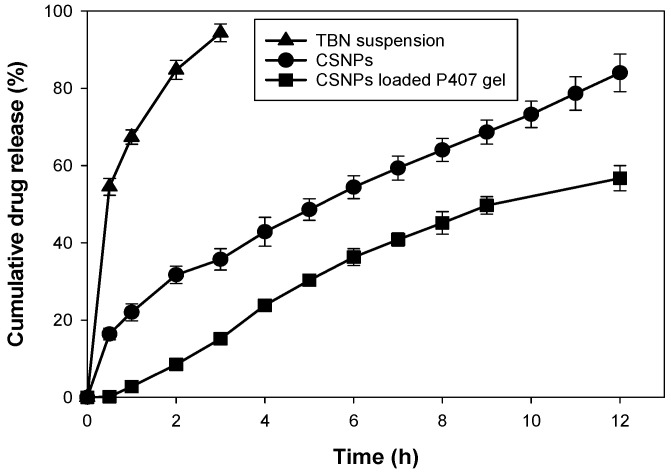
In vitro drug release profile of TBN-CSNP, TBN-CSNP loaded P407 gel and TBN suspension. Each value represent mean ± S.D (*n* = 3).

The percent cumulative release of TBN from TBN-CSNP suspension and TBN-CSNP loaded P407 gel are shown in Figure 8. The percent of TBN release from TBN-CSNPs was 84 ± 5% after 12 h in phosphate buffer (pH 5.5):ethanol. However, drug release from the TBN-CSNP-loaded P407 gel was extended as compared to nanoparticles released 57 ± 3% of TBN after 12 h. Drug release from drug suspension was 94 ± 2% after 3 h. Incorporation of TBN into CSNP significantly reduced the release of TBN as compared to the TBN suspension (*p* < 0.05), which was further reduced (*p* < 0.05) when CSNP was added into the P407 gel.

#### 3.5.5. Kinetic Modelling of In Vitro Drug Release

Table 3 shows the kinetic parameters of the drug release profile of optimized TBN-CSNP and TBN-CSNP loaded P407 gel. The drug release profile fitted best to the first-order kinetics (R^2^ = 0.9918) for both TBN-CSNP- and TBN-CSNP-loaded P407 gels, which showed that drug release was dependent on the concentration of drug remaining in the formulation. Korsmeyer–Peppas n value of 0.5 and 0.89 was observed for TBN-CSNP and TBN-CSNP loaded P407 gel, respectively. This depicts the anomalous behavior of drug release, which suggests that the release of drug from TBN-CSNP- and TBN-CSNP-loaded P407 gels occurs through both the diffusion and erosion of formulation.

#### 3.5.6. Ex Vivo Permeation Study

**Figure 9:**
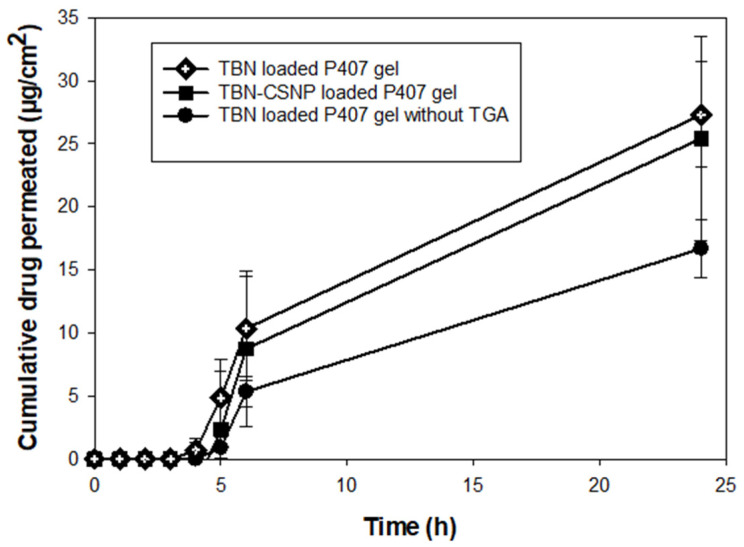
Ex vivo permeation across nail from TBN-CSNP loaded gel, TBN loaded P407 gel and TBN loaded gel (without TGA). Each value represent mean ± S.D (*n* = 3).

The ex vivo permeation (Figure 9) was studied by placing TBN-CSNP-loaded P407 gel, TBN-loaded P407 gel, and TBN-loaded P407 gel (without TGA) on the nail surface affixed between the donor and receiver compartment of the Franz diffusion cell. The concentration of TBN was observed to be increased with the increase in time. The cumulative quantity of TBN permeated from TBN-CSNP-loaded P407 gel, TBN-loaded P407 gel, and TBN-loaded P407 gel (without TGA) after 24h was 25 ± 8 µg/cm^2^, 27 ± 4, and 17 ± 2 µg/cm^2^, respectively. The permeated amount from TBN-CSNP-loaded P407 gel (25 ± 8 µg/cm^2^) was slightly lower than TBN-loaded P407 gel (27 ± 4), however, this difference was insignificant (*p* > 0.05).

#### 3.5.7. Nail Uptake Efficiency

The quantity of rhodamine B uptaken by nail was 3.6 ± 0.7 µg and 2.1 ± 0.7 µg following 2 h topical application of CSNPs loaded P407 gel and rhodamine B loaded P407 gel. When rhodamine B loaded P407 gel (without TGA) was applied for 2 h, then the quantity of rhodamine B taken by the nail was 1.6 ± 0.3 µg. Uptake of the rhodamine B was significantly greater (*p* < 0.05) when CSNP loaded P407 gel was used as compared to rhodamine B loaded P407 gel and rhodamine B loaded P407 gel (without TGA).

## 4. Discussion

The nanoparticles based topical drug delivery are advantageous because of their small size and direct application at the intended site [37]. Incorporation of polymeric nanoparticles into P407 gel improve colloidal stability of nanoparticles, reduce aggregation of nanoparticles and modulate the release of the drug [38]. In this study, polymeric CSNP were developed and incorporated into P407 gel to investigate its potential for transungual delivery of TBN.

Design of experiments is commonly applied to examine the impact of independent factors on responses of a process. The Design of experiments produces optimum response with minimum number of experiments. Central composite design is the widely design because of good predictability, minimum numbers of tests and high precision [39]. Quadratic model for both responses i.e., particle size and EE showed good fitting with the experimental values, as confirmed by high value of R^2^ and p value of less than 0.05. Pred R-squared and Adj R-squared values of models were close to each other. Furthermore, “Adequate precision” which evaluates signal to noise ratio was greater than 4 which indicates the model was adequate enough to predict responses.

Figures S1 and S3 (Supplementary Materials) depict the plots of predicted values versus actual values of particle size and EE respectively, which showed that predicted response was close to the experimental data. Each plot evaluates the capability of the model to predict response. Closer the predicted values to the actual values, closer will be the points to the line on the scatterplot and the model will offer good predictability. Less deviation in distribution of studentized residuals from the straight line was observed which confirmed low residual value and insignificant error (Figures S2 and S4 in Supplementary Materials). Further, CV of less than 10% showed the reproducibility of the suggested model. Lower value of CV in the current study (9.3% for particle size, 4.6% for EE) confirms the accuracy of the model (quadratic). Insignificant lack of fit value showed that quadratic model can be utilized to evaluate the impact of chitosan and tween 80 on particle size and EE.

Particle size was observed to be increase with increasing concentration of chitosan. This may be due to the high viscosity of the solution; thus less force is exerted under stirring of solution during preparation of nanoparticles [40]. Increase in viscosity of the organic phase also causes increase in the viscous forces which resist breakdown of the droplet producing bigger droplets, producing particles of greater size [41,42]. Higher the tween 80 concentration, smaller was the size of TBN-CSNP which may be due to the small droplet size of organic solvent produced during the formation of nanoparticles [43].

EE was increased with increase of chitosan concentration. Viscosity of the solution increases as concentration of the polymer is increased, this increases the thickness of diffusional pathway into the external aqueous phase, which reduces the loss of drug from organic droplets into the aqueous phase, and entrapping more quantity of drug in the polymeric nanoparticles [44]. Increase in particle size due to increasing quantity of polymer enhance entrapment of the drug by creating more space for entrapment of drug [43]. Another reason could be the availability of a greater amount of polymer to encapsulate the drug, thus not allowing saturation of polymer for encapsulation of drug [42]. Increasing concentration of tween 80 caused decrease of EE although its effect was insignificant. The decrease in the entrapment efficiency by increasing tween 80 concentrations may be due to the decreased droplet size during the formation of nanoparticles producing nanoparticle of smaller size and less entrapment of the drug [43].

The high magnitude of positive charge (+37 ± 1.5 mV) on the surface of prepared TBN-CSNP indicates good stability to prevent aggregation of nanoparticles [45]. Apart from colloidal stability, net surface charge of the nanoparticle formulation is also important parameter in terms of nanoparticle interaction with topical biological barriers and biological membranes inside the body. Electrostatic attraction between positively charged nanoparticles and negatively charged biological membranes favor uptake of nanoparticles [46]. Nail keratin has negative charge [47] which will attract the positively charged CSNP [48,49]. Positive charge will also help in easy penetration of CSNP in to the nail because of preferential diffusion of positively charged CSNP into the negatively charged keratin [50]. Stability study confirm that the final formulation was stable in terms of particle size, zeta potential and drug content at 5 ± 3 °C. Also, no significant changes were observed in these parameters of the TBN-CSNP when stored at 25 ± 2 °C and 40 ± 2 °C and retained the physicochemical characteristics like particle size, zeta potential and drug content.

The characteristic peaks of the TBN and Chitosan as previously described in the literature [51,52,53] were observed in the IR spectra of the formulated TBN-CSNP which established the absence of any polymer-drug interaction and confirm the entrapment of TBN in CSNP. XRD analysis indicated the reduction in the crystallinity of CSNP as compared to pure drug and chitosan. This reduction in crystallinity can be attributed to the crosslinking of the chitosan which may disrupt the intermolecular hydrogen bond [54,55].

The optimized formulation of TBN-CSNP was further utilized to prepare nail penetration enhancer (TGA) containing P407 gel formulation. Spreadability is an important property of topically applied formulations. Application of the topical formulation is more comfortable if the gel spreads easily [30]. CSNP loaded P407 gel showed pseudoplastic (non-Newtonian) behaviour. It means that application of force will decreases the viscosity of P407 gel on nail. This will help in attaining excellent spreadability of the gel as also evident from the good value of spreadability. Topical nail formulation should be retained for sufficient time when comes in contact with water and also can be easily removed with simple washing. Erosion profile of the prepared formulation showed that 60 ± 2% of the gel was remained on the surface while 40 ± 2% of gel was eroded after 12 washings.

TBN-CSNP showed initial burst release however, when nanoparticles were loaded in to P407 gel, no burst release was observed [56]. This may be attributed to the dissolution of chitosan in slight acidic pH of the media (pH 5.5) [57]. However, this was controlled when nanoparticles were loaded to P407 gel which further sustained the release of TBN as compared to the TBN-CSNP dispersion. TBN release from the TBN- CSNP occurred first and then diffused through the P407 gel matrix producing further sustained release effect [58].

It was observed that higher amount of TBN was permeated from TBN-CSNP loaded P407 gel and TBN loaded P407 gel as compared to TBN loaded P407 gel without TGA. A slight lower permeation from TBN-CSNP loaded P407 gel as compared to TBN loaded gel might occurred due to slow release of TBN from the TBN-CSNP. Although permeated drug concentration in the receiver fluid was above minimum inhibitory concentration (0.001–0.01 µg/mL) and minimal fungicidal concentrations (0.003–0.006 µg/mL) against dermatophytes [59], however antifungal study across nail plate need to be conducted to investigate the inhibitory effect of permeated drug. Rhodamine B dye was used to study the nail uptake efficiency due to its relatable physico-chemical properties, molecular weight (Mol. Wt. 443) with TBN and easiness in extraction from nails. The nail uptake study showed that greater uptake of the rhodamine was observed when TBN-CSNP loaded P407 gel formulation was allowed on the nail surface for 2 h as compared to TBN loaded P407 gel formulation which can be attributed to the electrostatic attraction between positively charged chitosan and negatively charged nail keratin [60]. These findings confirmed that TBN-CSNP can create drug reservoir in the nail plate, even if formulation resides for limited time on nail surface during performing routine life actives, which in case of conventional formulation will need frequent application for continuous supply of drug for permeation. Hence, developed TBN-CSNP loaded P407 gel formulation can be used as a potential carrier for transungual delivery of TBN for topical treatment of onychomycosis.

## 5. Conclusions

TBN-CSNP loaded P407 gel formulation was successfully formulated for transungual delivery of TBN to treat onychomycosis. Biodegradable chitosan was used because of its sustain release property and intrinsic antifungal activity against dermatophytes. Developed TBN-CSNP formulation exhibited good physicochemical characteristics and sustain release property. TBN-CSNP loaded P407 gel showed further sustain release property and improved TBN permeation across nail plate. CSNP loaded P407 gel also showed greater nail uptake efficiency as compared to TBN loaded P407 gel. Thus, prepared polymeric nanoparticles based topical formulation may be a potential candidate for the treatment of the onychomycosis to overcome the problem of frequent application of conventional formulation.

## Figures and Tables

**Table 1:** Different runs of TBN-CSNP formulations suggested by FCCCD and their responses (Expressed as mean ± S.D (*n* = 3)).

Code	Chitosan (% *w*/*v*)	Tween 80 (% *w*/*v*)	Particle Size (nm)	Entrapment Efficiency (%)
NP1	0.2	0.5	164 ± 9	56 ± 7
NP2	0.2	1	157 ± 16	55 ± 14
NP3	0.3	1	255 ± 72	78 ± 1
NP4	0.4	1	328 ± 30	80 ± 2
NP5	0.4	0.5	347 ± 16	77 ± 1
NP6	0.3	1	221 ± 1	81 ± 0.3
NP7	0.3	0.5	255 ± 37	74 ± 1
NP8	0.3	1	271 ± 16	71 ± 1
NP9	0.3	1.5	186 ± 40	76 ± 3
NP10	0.3	1	240 ± 1	79 ± 1
NP11	0.4	1.5	192 ± 31	75 ± 3
NP12	0.2	1.5	139 ± 2	49 ± 3
NP13	0.3	1	281 ± 53	76 ± 3

**Table 2:** Stability studies of TBN-CSNP at 5 ± 3 °C, 25 ± 2 °C and 40 ± 2 °C. Each value represents the mean ± S.D (*n* = 3).

Storage Temperature	Time Period (Months)	Particle Size (nm)	Zeta Potential (mV)	Drug Loading (%)
5 ± 3 °C	0	229 ± 5	37 ± 1.5	18 ± 1
	1	230 ± 5	37.1 ± 1	
	3	231 ± 6	37.0 ± 2	
	6	240 ± 8	37.5 ± 1.5	17 ± 1
25 ± 2°C	0	229 ± 5	37 ± 1.5	18 ± 1
	1	238 ± 10	39 ± 2	
	3	242 ± 11	40 ± 1.5	
	6	247 ± 12	40 ± 1	17 ± 1
40 ± 2 °C	0	229 ± 5	37 ± 1.5	18 ± 1
	1	241 ± 10	40 ± 1	
	3	253 ± 9	40 ± 2	
	6	253 ± 10	40 ± 2	16 ± 1

**Table 3:** Kinetic models for in vitro drug release.

Model		TBN-CSNP	TBN-CSNP Loaded P407 Gel
Zero order	K	7.834	5.37
	R^2^	0.7778	0.9660
First order	k1	0.139	0.071
	R^2^	0.9509	0.9784
Higuchi	kH	22.87	14.38
	R^2^	0.9881	0.8376
Korsmeyer peppas	kKP	21.65	6.65
	R^2^	0.9932	0.9693
	N	0.5	0.89

## Data Availability

Not applicable.

## Supplementary Materials

The following are available online at https://www.mdpi.com/article/10.3390/pharmaceutics14112353/s1, Figure S1: Design experts plot between predicted and actual values for Particle size; Figure S2: Normal plot of residuals for particle size; Figure S3: Design experts plot between predicted and actual values for EE; Figure S4: Normal plot of residuals for EE.

## References

[1] Hoda Q., Aqil M., Ahad A., Imam S.S., Praveen A., Qadir A., Iqbal Z. (2021). Optimization of Valencene Containing Lipid Vesicles for Boosting the Transungual Delivery of Itraconazole. 3 Biotech.

[2] Aggarwal R., Targhotra M., Sahoo P.K., Chauhan M.K. (2020). Onychomycosis: Novel Strategies for Treatment. J. Drug Deliv. Sci. Technol..

[3] Piraccini B.M., Alessandrini A. (2015). Onychomycosis: A Review. J. Fungi.

[4] Khattab A., Shalaby S. (2018). Optimized Ciclopirox-Based Eudragit RLPO Nail Lacquer: Effect of Endopeptidase Enzyme as Permeation Enhancer on Transungual Drug Delivery and Efficiency against Onychomycosis. AAPS PharmSciTech.

[5] Gupta A.K., Versteeg S.G., Shear N.H. (2017). Onychomycosis in the 21st Century: An Update on Diagnosis, Epidemiology, and Treatment. J. Cutan. Med. Surg..

[6] Barot B.S., Parejiya P.B., Patel H.K., Mehta D.M., Shelat P.K. (2012). Microemulsion-Based Antifungal Gel Delivery to Nail for the Treatment of Onychomycosis: Formulation, Optimization, and Efficacy Studies. Drug Deliv. Transl. Res..

[7] Fernández-Campos F., Navarro F., Corrales A., Picas J., Pena E., González J., Otero-Espinar F.J. (2020). Transungual Delivery, Anti-Inflammatory Activity, and In Vivo Assessment of a Cyclodextrin Polypseudorotaxanes Nail Lacquer. Pharmaceutics.

[8] Nair A.B., Kiran Vaka S.R., Murthy S.N. (2011). Transungual Delivery of Terbinafine by Iontophoresis in Onychomycotic Nails. Drug Dev. Ind. Pharm..

[9] Elsherif N.I., Shamma R.N., Abdelbary G. (2017). Terbinafine Hydrochloride Trans-Ungual Delivery via Nanovesicular Systems: In Vitro Characterization and Ex Vivo Evaluation. AAPS PharmSciTech.

[10] Yang F., Yu X., Shao W., Guo P., Cao S., Wang M., Wang Y., Wu C., Xu Y. (2020). Co-Delivery of Terbinafine Hydrochloride and Urea with an in Situ Film-Forming System for Nail Targeting Treatment. Int. J. Pharm..

[11] Nair A.B., Al-Dhubiab B.E., Shah J., Gorain B., Jacob S., Attimarad M., Sreeharsha N., Venugopala K.N., Morsy M.A. (2021). Constant Voltage Iontophoresis Technique to Deliver Terbinafine via Transungual Delivery System: Formulation Optimization Using Box–Behnken Design and In Vitro Evaluation. Pharmaceutics.

[12] Smith K.A., Hao J., Li S.K. (2011). Effects of Organic Solvents on the Barrier Properties of Human Nail. J. Pharm. Sci..

[13] Ullah K.H., Raza F., Munawar S.M., Sohail M., Zafar H., Zafar M.I., Ur-Rehman T. (2021). Poloxamer 407 Based Gel Formulations for Transungual Delivery of Hydrophobic Drugs: Selection and Optimization of Potential Additives. Polymers.

[14] Chiu W.S., Belsey N.A., Garrett N.L., Moger J., Price G.J., Delgado-Charro M.B., Guy R.H. (2015). Drug Delivery into Microneedle-Porated Nails from Nanoparticle Reservoirs. J. Control. Release.

[15] Wang F., Yang P., Choi J.S., Antovski P., Zhu Y., Xu X., Kuo T.H., Lin L.E., Kim DN H., Huang P.C. (2018). Cross-Linked Fluorescent Supramolecular Nanoparticles for Intradermal Controlled Release of Antifungal Drug-A Therapeutic Approach for Onychomycosis. ACS Nano.

[16] Soliman G.M. (2017). Nanoparticles as Safe and Effective Delivery Systems of Antifungal Agents: Achievements and Challenges. Int. J. Pharm..

[17] Dhamoon R.K., Popli H., Gupta M. (2019). Novel Drug Delivery Strategies for the Treatment of Onychomycosis. Pharm. Nanotechnol..

[18] Flores F.C., Rosso R.S., Cruz L., Beck RC R., Silva C.B. (2017). An Innovative Polysaccharide Nanobased Nail Formulation for Improvement of Onychomycosis Treatment. Eur. J. Pharm. Sci..

[19] Bhattarai N., Gunn J., Zhang M. (2010). Chitosan-Based Hydrogels for Controlled, Localized Drug Delivery. Adv. Drug Deliv. Rev..

[20] Ribeiro M.P., Espiga A., Silva D., Baptista P., Henriques J., Ferreira C., Silva J.C., Borges J.P., Pires E., Chaves P. (2009). Development of a New Chitosan Hydrogel for Wound Dressing. Wound Repair Regen..

[21] He W., Guo X., Xiao L., Feng M. (2009). Study on the Mechanisms of Chitosan and Its Derivatives Used as Transdermal Penetration Enhancers. Int. J. Pharm..

[22] Ta Q., Ting J., Harwood S., Browning N., Simm A., Ross K., Olier I., Al-Kassas R. (2021). Chitosan Nanoparticles for Enhancing Drugs and Cosmetic Components Penetration through the Skin. Eur. J. Pharm. Sci..

[23] Yien L., Zin N.M., Sarwar A., Katas H. (2012). Antifungal Activity of Chitosan Nanoparticles and Correlation with Their Physical Properties. Int. J. Biomater..

[24] Alqahtani A., Raut B., Khan S., Mohamed JM M., Fatease A.A.l., Alqahtani T., Alamri A., Ahmad F., Krishnaraju V. (2022). The Unique Carboxymethyl Fenugreek Gum Gel Loaded Itraconazole Self-Emulsifying Nanovesicles for Topical Onychomycosis Treatment. Polymers.

[25] Tayel S.A., El-Nabarawi M.A., Tadros M.I., Abd-Elsalam W.H. (2013). Positively Charged Polymeric Nanoparticle Reservoirs of Terbinafine Hydrochloride: Preclinical Implications for Controlled Drug Delivery in the Aqueous Humor of Rabbits. AAPS PharmSciTech.

[26] Saremi S., Atyabi F., Akhlaghi S.P., Ostad S.N., Dinarvand R. (2011). Thiolated Chitosan Nanoparticles for Enhancing Oral Absorption of Docetaxel: Preparation, in Vitro and Ex Vivo Evaluation. Int. J. Nanomed..

[27] Mulik R., Mahadik K., Paradkar A. (2009). Development of Curcuminoids Loaded Poly (Butyl) Cyanoacrylate Nanoparticles: Physicochemical Characterization and Stability Study. Eur. J. Pharm. Sci..

[28] Moore T., Croy S., Mallapragada S., Pandit N. (2000). Experimental Investigation and Mathematical Modeling of Pluronic(^®^) F127 Gel Dissolution: Drug Release in Stirred Systems. J. Control. Release.

[29] Chen P., Kohane D.S., Park Y.J., Bartlett R.H., Langer R., Yang V.C. (2004). Injectable Microparticle–Gel System for Prolonged and Localized Lidocaine Release. II. In Vivo Anesthetic Effects. J. Biomed. Mater. Res. Part A.

[30] Uprit S., Sahu R.K., Roy A., Pare A. (2013). Preparation and Characterization of Minoxidil Loaded Nanostructured Lipid Carrier Gel for Effective Treatment of Alopecia. Saudi Pharm. J..

[31] Khan A.W., Kotta S., Ansari S.H., Sharma R.K., Kumar A., Ali J. (2013). Formulation Development, Optimization and Evaluation of Aloe Vera Gel for Wound Healing. Pharmacogn. Mag..

[32] Iyer M.S., Gujjari A.K., Paranthaman S., Abu Lila A.S., Almansour K., Alshammari F., Khafagy E.-S., Arab H.H., Gowda D.V. (2022). Development and Evaluation of Clove and Cinnamon Supercritical Fluid Extracts-Loaded Emulgel for Antifungal Activity in Denture Stomatitis. Gels.

[33] Ur-Rehman T., Tavelin S., Gröbner G. (2011). Chitosan in Situ Gelation for Improved Drug Loading and Retention in Poloxamer 407 Gels. Int. J. Pharm..

[34] Tanrıverdi S.T., Özer Ö. (2013). Novel Topical Formulations of Terbinafine-HCl for Treatment of Onychomycosis. Eur. J. Pharm. Sci..

[35] Vaghasiya H., Kumar A., Sawant K. (2013). Development of Solid Lipid Nanoparticles Based Controlled Release System for Topical Delivery of Terbinafine Hydrochloride. Eur. J. Pharm. Sci..

[36] Baraldi A., Jones S.A., Guesné S., Traynor M.J., McAuley W.J., Brown M.B., Murdan S. (2015). Human Nail Plate Modifications Induced by Onychomycosis: Implications for Topical Therapy. Pharm. Res..

[37] Sohail M., Rabbi F., Younas A., Hussain A., Yu B., Li Y., Iqbal S., Ullah K.H., Qadeer A., Aquib M. (2022). Herbal Bioactive–Based Nano Drug Delivery Systems. Herbal Bioactive-Based Drug Delivery Systems.

[38] Grillo R., Dias F.V., Querobino S.M., Alberto-Silva C., Fraceto L.F., de Paula E., de Araujo D.R. (2019). Influence of Hybrid Polymeric Nanoparticle/Thermosensitive Hydrogels Systems on Formulation Tracking and in Vitro Artificial Membrane Permeation: A Promising System for Skin Drug-Delivery. Colloids Surf. B Biointerfaces.

[39] Elsayed E.W., El-Ashmawy A.A., Mursi N.M., Emara L.H. (2019). Optimization of Gliclazide Loaded Alginate-Gelatin Beads Employing Central Composite Design. Drug Dev. Ind. Pharm..

[40] Esmaeilzadeh-Gharedaghi E., Faramarzi M.A., Amini M.A., Rouholamini Najafabadi A., Rezayat S.M., Amani A. (2012). Effects of Processing Parameters on Particle Size of Ultrasound Prepared Chitosan Nanoparticles: An Artificial Neural Networks Study. Pharm. Dev. Technol..

[41] Budhian A., Siegel S.J., Winey K.I. (2005). Production of Haloperidol-Loaded PLGA Nanoparticles for Extended Controlled Drug Release of Haloperidol. J. Microencapsul..

[42] Sharma N., Madan P., Lin S. (2016). Effect of Process and Formulation Variables on the Preparation of Parenteral Paclitaxel-Loaded Biodegradable Polymeric Nanoparticles: A Co-Surfactant Study. Asian J. Pharm. Sci..

[43] Krishnamachari Y., Madan P., Lin S. (2007). Development of PH-and Time-Dependent Oral Microparticles to Optimize Budesonide Delivery to Ileum and Colon. Int. J. Pharm..

[44] Jyothi NV N., Prasanna P.M., Sakarkar S.N., Prabha K.S., Ramaiah P.S., Srawan G.Y. (2010). Microencapsulation Techniques, Factors Influencing Encapsulation Efficiency. J. Microencapsul..

[45] Bseiso E.A., Nasr M., Sammour O.A., Abd El Gawad N.A. (2016). Novel Nail Penetration Enhancer Containing Vesicles “NPEVs” for Treatment of Onychomycosis. Drug Deliv..

[46] Baspinar Y., Borchert H.-H. (2012). Penetration and Release Studies of Positively and Negatively Charged Nanoemulsions—Is There a Benefit of the Positive Charge?. Int. J. Pharm..

[47] DeFrates K., Markiewicz T., Gallo P., Rack A., Weyhmiller A., Jarmusik B., Hu X. (2018). Protein Polymer-Based Nanoparticles: Fabrication and Medical Applications. Int. J. Mol. Sci..

[48] Desai P., Patlolla R.R., Singh M. (2010). Interaction of Nanoparticles and Cell-Penetrating Peptides with Skin for Transdermal Drug Delivery. Mol. Membr. Biol..

[49] Vejnovic I., Simmler L., Betz G. (2010). Investigation of Different Formulations for Drug Delivery through the Nail Plate. Int. J. Pharm..

[50] Baswan S.M., Li S.K., LaCount T.D., Kasting G.B. (2016). Size and Charge Dependence of Ion Transport in Human Nail Plate. J. Pharm. Sci..

[51] Pervaiz F., Mushtaq R., Noreen S. (2021). Formulation and Optimization of Terbinafine HCl Loaded Chitosan/Xanthan Gum Nanoparticles Containing Gel: Ex-Vivo Permeation and in-Vivo Antifungal Studies. J. Drug Deliv. Sci. Technol..

[52] Elmizadeh H., Khanmohammadi M., Ghasemi K., Hassanzadeh G., Nassiri-Asl M., Garmarudi A.B. (2013). Preparation and Optimization of Chitosan Nanoparticles and Magnetic Chitosan Nanoparticles as Delivery Systems Using Box–Behnken Statistical Design. J. Pharm. Biomed. Anal..

[53] Li P., Wang Y., Peng Z., She F., Kong L. (2011). Development of Chitosan Nanoparticles as Drug Delivery Systems for 5-Fluorouracil and Leucovorin Blends. Carbohydr. Polym..

[54] Dammak I., Bittante AM Q.B., Lourenco R.V., do Amaral Sobral P.J. (2017). Properties of Gelatin-Based Films Incorporated with Chitosan-Coated Microparticles Charged with Rutin. Int. J. Biol. Macromol..

[55] Mi F.-L., Huang C.-T., Liang H.-F., Chen M.-C., Chiu Y.-L., Chen C.-H., Sung H.-W. (2006). Physicochemical, Antimicrobial, and Cytotoxic Characteristics of a Chitosan Film Cross-Linked by a Naturally Occurring Cross-Linking Agent, Aglycone Geniposidic Acid. J. Agric. Food Chem..

[56] Liu H., Li W., Liu C., Tan J., Wang H., Hai B., Cai H., Leng H.-J., Liu Z.-J., Song C.-L. (2016). Incorporating Simvastatin/Poloxamer 407 Hydrogel into 3D-Printed Porous Ti6Al4V Scaffolds for the Promotion of Angiogenesis, Osseointegration and Bone Ingrowth. Biofabrication.

[57] Fan M., Hu Q., Shen K. (2009). Preparation and Structure of Chitosan Soluble in Wide PH Range. Carbohydr. Polym..

[58] Wen Y., Ban J., Mo Z., Zhang Y., An P., Liu L., Xie Q., Du Y., Xie B., Zhan X. (2018). A Potential Nanoparticle-Loaded in Situ Gel for Enhanced and Sustained Ophthalmic Delivery of Dexamethasone. Nanotechnology.

[59] Darkes MJ M., Scott L.J., Goa K.L. (2003). Terbinafine: A Review of Its Use in Onychomycosis in Adults. Am. J. Clin. Dermatol..

[60] Wu X., Landfester K., Musyanovych A., Guy R.H. (2010). Disposition of Charged Nanoparticles after Their Topical Application to the Skin. Skin Pharmacol. Physiol..

