# Supplementary material for: Chitosan Nanoparticles Loaded Poloxamer 407 Gel for Transungual Delivery of Terbinafine HCl
Source: Pharmaceutics. 2022 Oct 31;14(11):2353. doi: 10.3390/pharmaceutics14112353 (PMC9698022; doi:10.3390/pharmaceutics14112353)
Supplement: Supplementary file 1 [file pharmaceutics-14-02353-s001.zip › pharmaceutics-1941474-Supplementary.pdf]

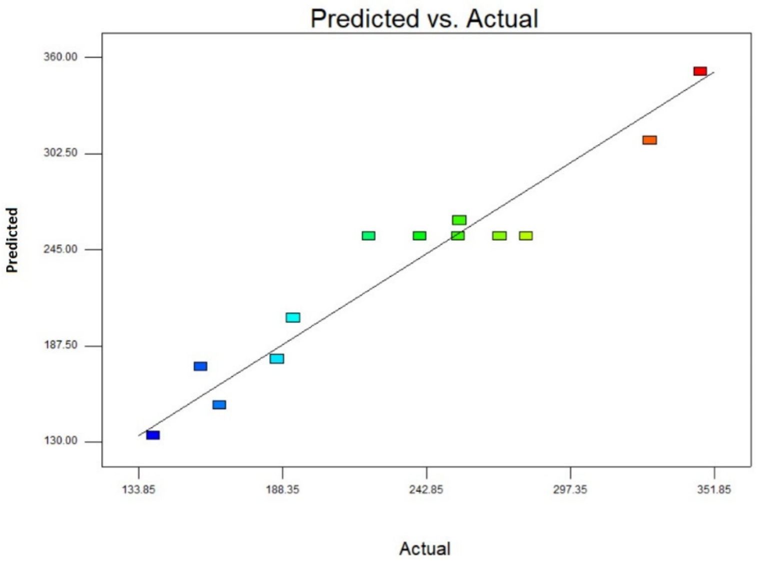

Figure S1. Design experts plot between predicted and actual values for particle size.

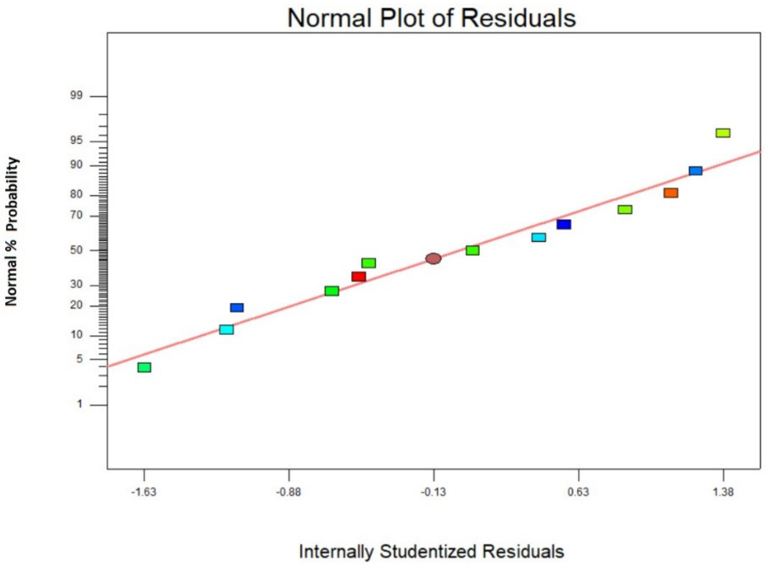

Figure S2. Normal plot of residuals for particle size.

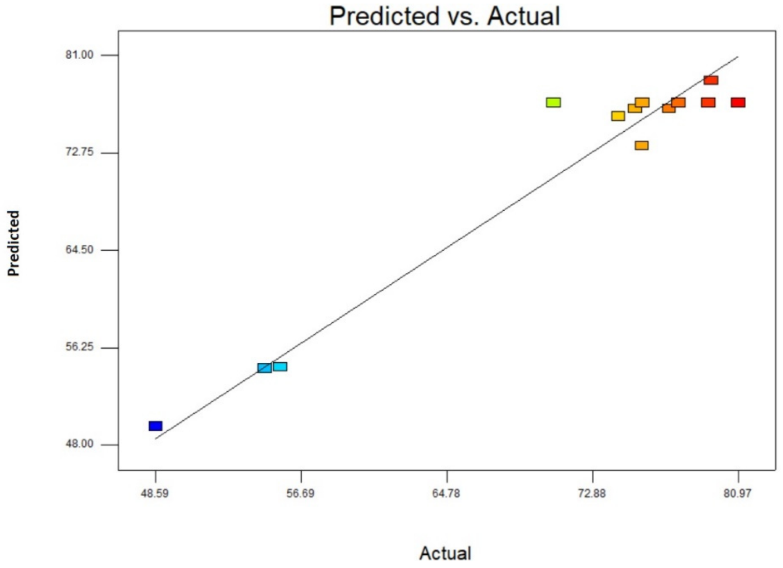

Figure S3. Design experts plot between predicted and actual values for EE.

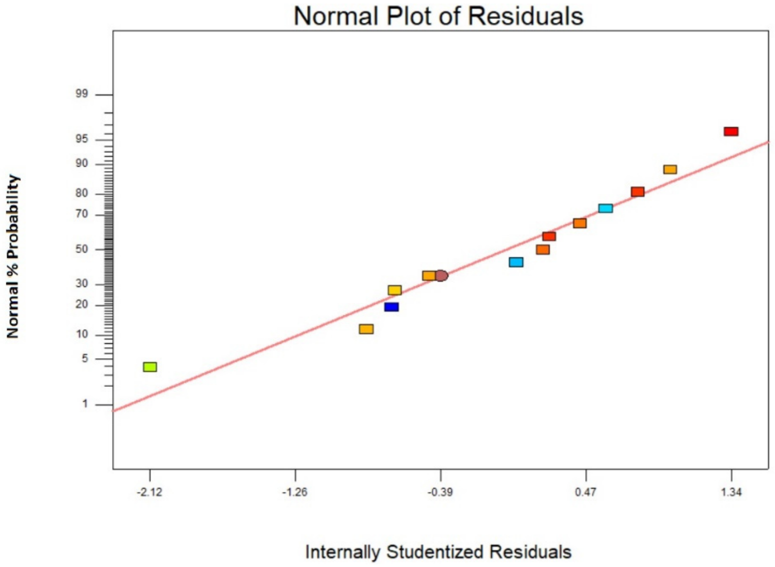

Figure S4. Normal plot of residuals for EE.
